# Eating Pork

**Published:** 1851-11

**Authors:** 


*Eating Pork.* — The New Hampshire Shakers have abandoned pork, as food, and they are not without good reasons for doing so. Moses understood the injurious effects of swine's flesh, which he learned of the Egyptians, and therefore interdicted it in his judicial character. The Egyptian priests, who were both philosophers and physicians, ages upon ages before the birth of the Jewish law-giver, had gained an insight into the constitution of man, which, transmitted through the Israelites, diluted and corrupted as it may be, still exerts a powerful influence on all modern systems of legislation.

Whatever was unclean in the Mosaic catalogue of edibles, is still thought to be unfit for human food, with the exception of swine. It is an anomaly that the one article, more abhorred than all others in the Levitical code, should become a favorite dish with the American Gentiles. Scrofulous affections, if not generated, are thought to be aggravated by pork; and the measles has been charged to its use. The hog is omnivorous, and more uncleanly than any other animal domesticated for economical purposes — a fact in itself sufficiently strong to deter the Jews from using the meat. Let those who are possessed of the information, show how much more we suffer from certain cutaneous and glandular diseases than the people of countries where pork is not used for food. We never saw a single swine in the whole of Egypt or Syria. The old prejudice, or the ancient interdiction, appears to influence the public sentiment in those countries. With these views, we doubt not that the Shakers will be gainers in health, and perhaps in longevity, by eschewing pork. Lard oil and stearine, in domestic economy, are invaluable articles; and when the demand for them requires all the swine raised, it will be a happy circumstance for the people. — *Boston Medical and Surgical Journal Editorial.*

